# Supplementary material for: Biomarkers of Neurodegeneration in Post-Traumatic Stress Disorder: An Integrative Review
Source: Biomedicines. 2023 May 17;11(5):1465. doi: 10.3390/biomedicines11051465 (PMC10216622; doi:10.3390/biomedicines11051465)
Supplement: Supplementary file 1 [file biomedicines-11-01465-s001.zip › biomedicines-2345127-supplementary.pdf]

Supplementary Table S1: Search strings for the current review, with numerical results for the PubMed database

String	Number of citations retrieved
1. "post-traumatic stress disorder" OR "posttraumatic stress disorder" OR "PTSD"	45752
2. "neurodegeneration" OR "neurodegenerative" OR "Alzheimer's disease" OR "Alzheimer's dementia" OR "dementia" OR "Parkinson's disease" OR "Parkinsonism"	468251
3. "biomarker" OR "biomarkers" OR "biological marker" OR "biological markers"	728059
4. "genetic" OR "genetics" OR "epigenetic" OR "epigenetics"	4794935
5. "immune" OR "immunological" OR "inflammation" OR "inflammatory" OR "immune-inflammatory"	2150684
6. "amyloid" OR "amyloid beta" OR "tau protein"	121140
7. "endocrine" OR "neuroendocrine"	262258
8. "imaging" OR "neuroimaging" OR "magnetic resonance imaging" OR "functional magnetic resonance imaging" OR "diffusion tensor imaging" OR "positron emission tomography" OR "single photon emission computerized tomography" OR "MRI" OR "fMRI" OR "DTI" OR "SPECT"	1172740
9. 1 AND 2	818
10. 9 AND (3 OR 4 OR 5 OR 6 OR 7 OR 8)	160
